# Supplementary material for: Conditional chemoconnectomics (cCCTomics) as a strategy for efficient and conditional targeting of chemical transmission
Source: eLife. 2024 Apr 30;12:RP91927. doi: 10.7554/eLife.91927 (PMC11060718; doi:10.7554/eLife.91927)
Supplement: Figure 3—source data 3. [file elife-91927-fig3-data3.zip › Figure 3 source data3 part 1.zip!/Figure 3-Source data 3-part1/1081-1-CRISPResso_Report_8zosk6yj.zip!/Nucleotide_percentage_quilt_around_sgRNA_TTTCTGGACTACAGTCCCCA.pdf]

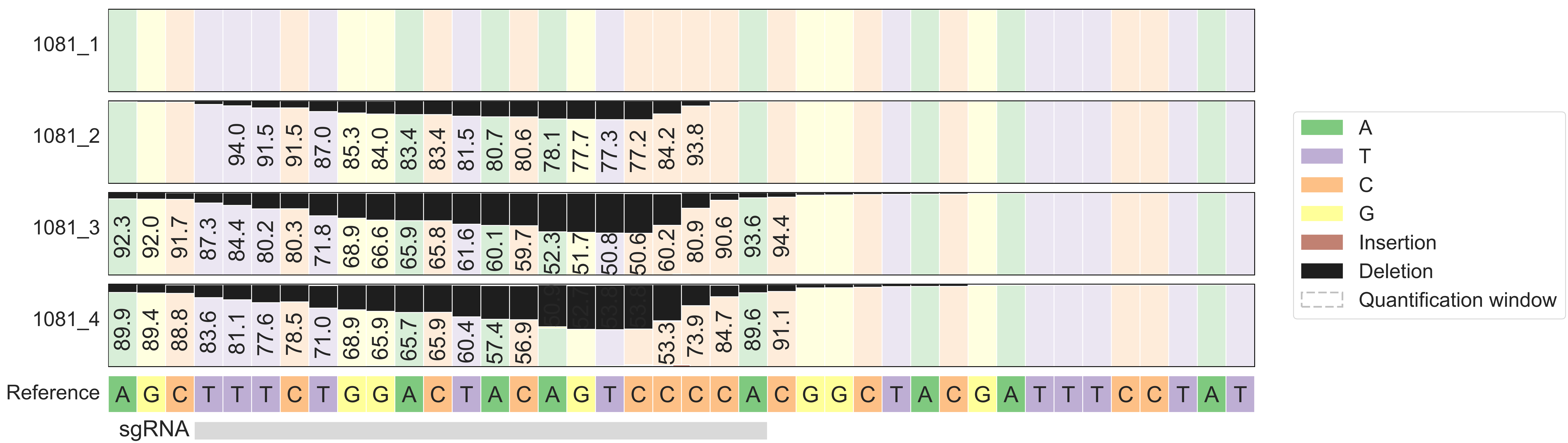

1081_1
1081_2
94.0 91.5 91.5 87.0 85.3 84.0 83.4 83.4 81.5 80.7 80.6 78.1 77.7 77.3 77.2 84.2 93.8
1081_3
92.3 92.0 91.7 87.3 84.4 80.2 80.3 71.8 68.9 66.6 65.9 65.8 61.6 60.1 59.7 52.3 51.7 50.8 50.6 60.2 80.9 90.6 93.6 94.4
1081_4
89.9 89.4 88.8 83.6 81.1 77.6 78.5 71.0 68.9 65.9 65.7 65.9 60.4 57.4 56.9 53.3 73.9 84.7 89.6 91.1
Reference
A G C T T T C T G G A C T A C A G T C C C C A C G G C T A C G A T T T C C T A T
sgRNA
A
T
C
G
Insertion
Deletion
Quantification window
